# Preparation and Properties of Electro-Blown Spinning Erythritol-Based Coaxial Phase Change Fibers

**DOI:** 10.3390/polym18080923

**Published:** 2026-04-09

**Authors:** Jiaxi Yang, Bingnan Chen, Yanxiong Qiao, Zhiguo Ma, Chuanxi Qiao, Zehao Wang, Heqiang Zheng, Zhiqiang Bian, Na Huang, Chunguang Wei, Jun Liu, Ding Nan

**Affiliations:** 1 Northern United Power Co., Ltd., Hohhot 010020, China; 2 School of Renewable Energy, Inner Mongolia University of Technology, Erdos 017010, China; 3 School of Materials Science and Engineering, Inner Mongolia University of Technology, Hohhot 010051, Chinaclxylj74@imut.edu.cn (J.L.); 4 Xilingol Thermal Power Plant of Northern United Power Co., Ltd., Xilingol 026000, China; 5 Dalat Power Plant of Northern United Power Co., Ltd., Ordos 017010, China; 6 College of Chemistry and Chemical Engineering, Inner Mongolia University, Hohhot 010021, China

**Keywords:** electro-blown spinning, erythritol, coaxial fibers, phase change materials, thermal energy storage

## Abstract

Phase change thermal storage fibers with high latent heat have attracted significant attention in thermal management and heat storage. Through fiber encapsulation, shape-stable phase change materials can be prepared, thereby expanding their applications. In this study, electro-blown spinning was utilized to prepare phase change materials (PCM) using erythritol, with polyethylene oxide (PEO) as the carrier material. Coaxial thermal storage fibers encapsulating the phase change materials were prepared using polyvinyl alcohol (PVA) and polyvinylpyrrolidone (PVP). The results indicate that the composite fibers have a smooth surface, uniform and smooth morphology, a maximum latent heat of 223.01 J/g, as well as excellent thermal stability. The coaxial fibers exhibit a distinct core–shell structure, with the coaxial fibers encapsulated with PVA as the shell material, demonstrating a high latent heat of 118.62 J/g, a residual rate of 93.81% after heating, and excellent thermal performance. The encapsulation efficiency is 53%, effectively addressing the issue of erythritol leakage. The research results provide valuable guidance for the efficient preparation of erythritol coaxial thermal storage fibers.

## 1. Introduction

Phase change thermal storage is now a leading energy storage technology, thanks to its distinct benefits including environmental friendliness and low cost [1]. As the core of phase change thermal storage, phase change materials (PCMs) absorb and store heat energy at the phase change temperature, before releasing a large amount of latent heat at the critical phase change temperature [2]. PCMs have excellent properties such as high heat capacity, thermal stability, and environmental friendliness [3]. However, the low thermal conductivity, high degree of supercooling, and leakage issues of PCM severely restrict further development. To alleviate the leakage problem, current research efforts are focused on developing new encapsulation strategies. Shape-stable PCM can be prepared using various methods, such as porous material loading method [4], microencapsulation method [5], and electrospinning encapsulation method [6]. Among them, electrospinning is a simple, convenient, and economical technique for preparing ultrafine (submicron) fibers [7]. The working principle is to apply a charge to a polymer solution in order to overcome surface tension [8]. Due to electrostatic repulsion, the polymer solution flows towards the grounded collector, and as the solvent evaporates from the solution [9], the fibers randomly deposit onto the collector. However, electrospinning has three main disadvantages: (1) The electrospinning process requires extremely high voltage, which can lead to safety issues [10]. (2) The feeding rate of the injection pump is low, resulting in low productivity [11]. (3) The materials for electrospinning must have high conductivity, which limits the selection of materials [12].

Emerging spinning methods include centrifugal jet spinning [13], plasma-induced synthesis [14], and solution blow spinning (SBS) [15]. In addition, microfiber preparation via matrix extraction has also been reported, where target fibers are obtained by removing a sacrificial matrix phase [16]. Among them, SBS produces nanofibers from polymer solutions using pressurized gas. The polymer solution is in the inner axis, and the pressurized gas is in the outer axis. The pressurized high-speed gas blown from the outer axis of the nozzle causes pressure drop and shearing at the gas/solution interface, stretching the polymer solution towards a fixed collector. Then, as the solvent evaporates, the stretched polymer forms fibers [17]. SBS solves the problems of poor safety and low productivity of electrostatic spinning, while the materials can be synthetic or bio-based polymers, increasing the variety of fiber materials [18]. However, traditional SBS processes also have some drawbacks [19], such as uncontrolled deposition of nanofibers [20], nozzle clogging [16], and jet instability [21], etc.

Electro-blown spinning (EBS) [22] combines the advantages of electrospinning and SBS. Adding an electric field in the traditional SBS process significantly enhances the stretching and traction of the jet [23], with the electric field voltage being generally less than 5 kV, which corresponds to a maximum current of 25 mA, much lower than that applied by electrostatic spinning. The synergistic effect of the electric field and compressed airflow provides excellent efficiency for fiber deposition [24], improving the jet stability of SBS. It also increases the spinning rate, overcoming the low productivity limitation of electrospinning. Ju et al. [25] created honeycomb-like porous structures of PVA/PTFE carbon fibers, which are suitable for adsorption, ion exchange, and catalyst carriers. Liu et al. [26] developed freestanding polyamide (PA-66) membranes for filtering aerosol particles. Zhou et al. [23] produced CeO_2_/CuO/Al_2_O_3_ ultrafine fibers that exhibited excellent catalytic efficiency in dye degradation, with enhanced solar energy utilization through CuO loading. However, using these methods to prepare composite fibers does not fully prevent materials from being exposed to the environment. Coaxial fibers [27] with core–shell structures have gained significant interest among researchers due to their ability to load and encapsulate high amounts of materials. This method involves connecting separate channels of different solutions to a concentrically arranged nozzle, producing fibers with a core–shell structure. The shell solution provides protection and encapsulation, preventing the core solution from coming into direct contact with the environment [28]. Lu et al. [29] conducted a study where they used coaxial electrospinning to create phase change heat storage fibers. They utilized PAN as the shell material and paraffin as the core material. On the other hand, Hu et al. [30] employed the same technique to fabricate phase change heat storage fibers. Their approach involved using polyurethane as the shell and soy wax as the core. Finally, Zhou et al. [31] produced flexible hollow CeO_2_/Al_2_O_3_ fibers for adsorption by combining coaxial electrospinning and air spinning techniques.

**Figure 1:**
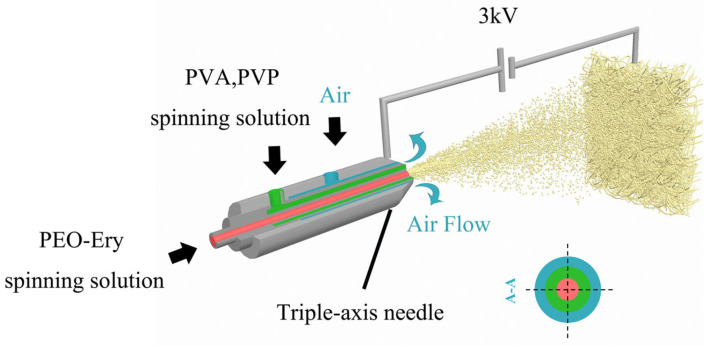
Schematic diagram of coaxial electro-blown spinning in this experiment.

Despite the wide application of coaxial nanofibers in many fields, there is currently limited literature on the manufacturing process, structure, and thermal performance of coaxial phase change fibers used for thermal energy storage. In particular, there are very few studies on coaxial phase change fibers prepared using EBS. Additionally, erythritol itself is not easily spinnable and is soluble in water and slightly soluble in organic solvents, making it difficult to encapsulate using spinning technology. In this study, polyethylene oxide (PEO) loaded with erythritol was selected to prepare PEO-Ery composite fibers as the core material. Polyvinyl alcohol (PVA) and polyvinylpyrrolidone (PVP) were selected as shell materials for the comparison of encapsulation materials. The coaxial phase change thermal storage fiber was prepared by the process of electro-blown spinning, as shown in Figure 1 below. Comparing the two kinds of shell materials, the one with the best comprehensive performance was selected to solve the leakage problem of erythritol in the phase change process. Meanwhile, the spinning efficiency was improved to provide a way for the industrialization of erythritol thermal storage fibers. Therefore, the objective of this study is to fabricate erythritol-based coaxial phase change fibers via electro-blown spinning, as well as to systematically investigate the effects of shell materials (PVA and PVP) on fiber morphology, encapsulation efficiency, and thermal performance.

## 2. Materials and Methods

### 2.1. Materials

Polyvinyl alcohol (PVA1788, Mw = 80,000, purchased from Hefei Sipin, Hefei, China). Polyvinylpyrrolidone (PVP10, Mw = 1,300,000, purchased from Shanghai Ball Chemical Reagent Co., Shanghai, China). PVA and PVP were selected as shell materials due to their good film-forming ability, water solubility, and compatibility with PEO. The molecular weights were chosen to ensure sufficient viscosity for stable fiber formation and effective encapsulation. Polyethylene oxide (PEO, Mw = 100,000, purchased from RYOJI, Dusseldorf, Germany). Erythritol is a filler-type sweetener with a melting point of 118 °C and a latent heat of 282 J/g, purchased from Shandong Baolingbao Biological Co., Yucheng, China. All the reagents were analytical reagents and were not purified further.

### 2.2. Preparation of PEO-Ery Uniaxial Fibers

The electro-blown spinning (EBS) setup used in this study consists of a coaxial needle system, two independent syringe pumps, a high-voltage power supply, and a compressed air supply system. The inner and outer channels are used to deliver the core solution and high-speed aerodynamic flow, respectively. The combined action of the electric field and high-speed airflow enables effective stretching and deposition of the fibers. A 1 g sample of PEO was dissolved into 9 mL of ultrapure water to equip a 10 wt.% aqueous solution of PEO. When the PEO was completely dissolved, 1 g, 1.5 g, 2.33 g, and 4 g of erythritol were added to the aqueous solution of PEO, respectively, and stirred for 3 h at 150 r/min to prepare the PEO spinning solution loaded with 50%, 60%, 70%, and 80% erythritol. The loading amount of erythritol is calculated by the following Formula (1). The solution was then injected into a 20 mL plastic syringe and spun with a coaxial needle, with the inner shaft being a 17-gauge needle (inner diameter: 1.07 mm, outer diameter: 1.47 mm) and the outer shaft being a 13-gauge needle (inner diameter: 1.90 mm, outer diameter: 2.40 mm). The inner shaft delivered the PEO-Ery spinning solution at a rate of 3 mL/h, while the outer shaft emitted a high-speed airflow of 0.35 MPa and 15 LPM. A metal mesh receiver was positioned 30 cm from the needle, and a voltage of 3 kV was applied between the tip of the coaxial needle and the receiver, causing the blended fibers to deposit on the metal mesh, thus preparing the PEO-Ery composite phase change fibers.

(1) $$A=\frac{B}{B+C}$$

where A is the loading of erythritol, B is the addition of erythritol, and C is the addition of PEO.

### 2.3. Preparation of PVA, PVP/PEO-Ery Coaxial Fiber

A triple-channel coaxial nozzle was employed to ensure stable formation of the core–shell structure, where the inner channel delivers the core solution, the middle channel delivers the shell solution, and the outer channel provides high-speed airflow for jet stretching. PVA, PVP was selected as the shell material for coaxial fibers, with 1 g PVA, PVP dissolved into 9 mL of ultrapure water and stirred at 150 r/min for 5 h to equip 10 wt.% PVA, PVP spinning solution as the shell material. PEO-80% Ery was selected as the core material and prepared as described in Section 2.2. Inject the solution into two 20 mL plastic syringes, and then use two independent injection pumps to inject the solution into the triple-axis needle. The inner shaft is a 17-gauge needle (inner diameter: 1.07 mm, outer diameter: 1.47 mm), the middle shaft is a 13-gauge needle (inner diameter: 1.90 mm, outer diameter: 2.40 mm), and the outer shaft is a 10-gauge needle (inner diameter: 3.00 mm, outer diameter: 3.53 mm). The inner shaft delivers the PEO-80% Ery spinning solution at a rate of 3 mL/h, the middle shaft propels the PVA and PVP spinning solution at a rate of 5 mL/h, and the outer shaft outputs a high-pressure airflow of 0.35 MPa and 20 LPM. The metal mesh receiver is positioned 30 cm from the needle, and a voltage of 3 kV is applied between the tip of the coaxial needle and the receiver, causing the blended fibers to deposit on the metal mesh, thus preparing two different coaxial phase change fibers, PVA/PEO-80% Ery and PVP/PEO-80% Ery, with different sheath materials.

### 2.4. Characterization

The solution properties consisted of viscosity and surface tension, which were determined by a viscometer (Brookfield DV2TR, Böhlerfeld, Elgin, IL, USA) using a SC4-21 rotor at 50 rpm and a surface tension meter (BZY-A, Shanghai Fangrui Instrument Co., Ltd., Shanghai, China). All measurements are conducted at 25 °C and the average values are obtained from at least three repeated measurements. The phase structure, composition, and crystallinity of the materials were analyzed by X-ray diffraction (XRD, D/MAX-2500/PC, Rigaku, Tokyo, Japan) at 2°/min with scanning angles from 10° to 50°. The functional groups of the fibers were analyzed by Fourier transform infrared spectroscopy (FT-IR, TENSOR II, Bruker, Bremen, Germany). The thermal performance of the fibers is determined using a differential scanning calorimeter (DSC, STA449F3, Netzsch, Selb, Germany) at a heating rate of 5 °C/min from room temperature to 200 °C, under a nitrogen gas (N_2_) flow rate of 50 mL/min. The thermal stability of the fibers is measured using a thermogravimetric analyzer (TGA, STA-449 F3, Netzsch, Selb, Germany) at a heating rate of 5 °C/min from room temperature to 200 °C, under a nitrogen gas (N_2_) flow rate of 50 mL/min. Characterization of fiber morphology by field emission ambient scanning electron microscopy (SEM, HITACHI-SU8220, Hitachi, Tokyo, Japan). Prior to SEM testing, the samples are coated with a thin layer of gold using a sputter coater. SEM images are taken at an accelerating voltage of 20 kV. The fiber diameter is measured from the SEM images using Image J 6.0 software. The average nanofiber diameter is calculated based on at least 50 measurements per sample, and the corresponding standard deviation is included to reflect the diameter distribution. The structure of the fibers is characterized using a field emission transmission electron microscope (TEM, JEM-2010, JEOL, Tokyo, Japan).

## 3. Results and Discussion

### 3.1. Characterization of Fibers

**Figure 2:**
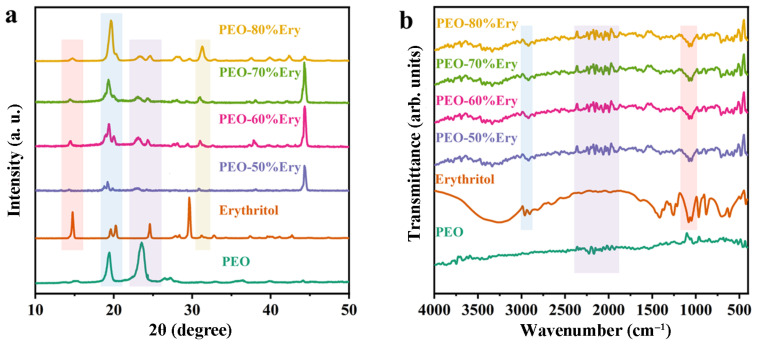
(**a**) XRD pattern. (**b**) Fourier transform infrared (FT-IR) spectrum.

The phase structure of the fibers was analyzed using X-ray diffractometry, as shown in Figure 2a below. It can be observed that erythritol exhibits distinct diffraction peaks at 2θ = 14°, 20°, 24°, and 29°. PEO shows prominent diffraction peaks at 2θ = 19° and 23.5°. In the composite fiber, the diffraction peak at 14° corresponds to the erythritol peak, while the three peaks appearing at 19° and 20° correspond to two erythritol peaks and one PEO peak. The two peaks within the range of 17.5–25° correspond to the erythritol and PEO peaks, respectively. The diffraction peaks in the composite fiber are the superposition of the diffraction peaks of PEO and erythritol, and no new diffraction peaks appear, indicating that the different components are only physically combined and no chemical reaction occurred. The intensity of the diffraction peaks in the composite fiber is lower compared to that of erythritol and PEO. This may be attributed to the higher crystal defects in the composite material, which scatter and lose X-rays, thereby reducing the peak intensity. Alternatively, it could be due to the interaction between X-rays and the different densities or atomic numbers of the components in the composite material, resulting in reduced intensity.

The changes in functional groups of the fibers were analyzed using Fourier transform infrared spectroscopy (FT-IR), as shown in Figure 2b. The peak of erythritol at 3400–3200 cm^−1^ corresponds to the stretching vibration of -OH, the peak at 2900 cm^−1^ corresponds to the stretching vibration of -CH_2_, -CH, the peak at 1650 cm^−1^ corresponds to the stretching vibration of C=O, while the peak at 1400 cm^−1^ represents the in-plane bending vibration of -OH, the peak at 1200–1000 cm^−1^ corresponds to the C-O-C vibration, and the peak at 825 cm^−1^ is the characteristic absorption of the C-C single bond of alcohols. The peak near 3460 cm^−1^ for PEO corresponds to the stretching vibration of -OH, the peak near 2980–2880 cm^−1^ corresponds to the stretching vibration of -CH, the peak near 1460 cm^−1^ corresponds to the bending vibration of -CH, and the peak near 1100–1050 cm^−1^ corresponds to the C-O-C stretching vibration. As can be seen from the figure, the infrared spectrum of PEO-Ery is the superposition of the respective characteristic absorption peaks of PEO and erythritol, and no new peaks appeared, indicating that the chemical groups of the composite fiber originated from the mixing of the substances, and no new substances were generated.

### 3.2. Microscopic Morphology Analysis of Fibers

**Figure 3:**
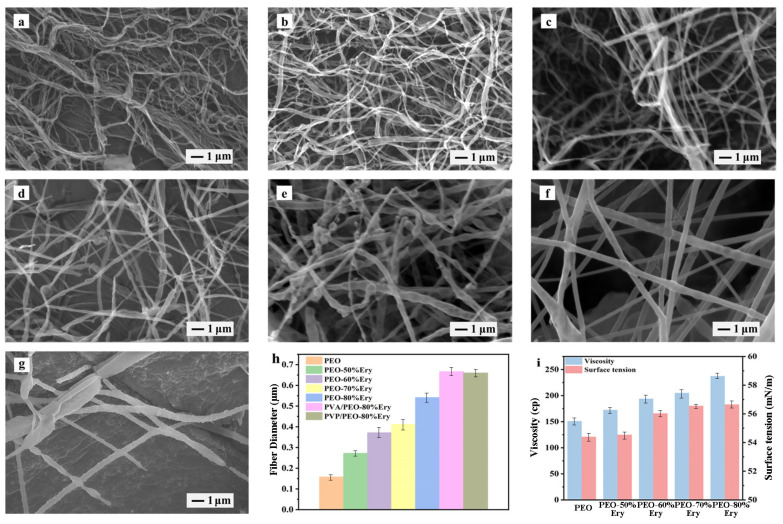
SEM images of fibers: (**a**) PEO, (**b**) PEO-50%Ery, (**c**) PEO-60%Ery, (**d**) PEO-70%Ery, (**e**) PEO-80%Ery, (**f**) PVA/PEO-80%Ery, and (**g**) PVP/PEO-80%Ery. (**h**) Average fiber diameter with standard deviation (*n* ≥ 50). (**i**) The viscosity and surface tension of the spinning solution.

Field emission environmental scanning electron microscopy is used to observe the surface morphology of fibers. The SEM image of PEO fibers is shown in Figure 3a, with a cylindrical fiber morphology, smooth surface, uniform structure, and an average diameter of 0.158 μm. The SEM images of composite fibers loaded with 50%, 60%, 70%, and 80% Ery are shown in Figure 3b–e. By comparison, it is found that after adding erythritol, the overall fiber morphology remains unchanged, while the high viscosity of the sugar alcohol material causes the fibers to appear as interconnected network. With the increase in erythritol content, the fiber diameter becomes thicker, as shown in Figure 3h, mainly due to the influence of the solution’s viscosity and surface tension. Specifically, the increase in erythritol content significantly enhances the viscosity of the spinning solution, leading to stronger molecular chain entanglement and reduced stretching of the jet under the combined electric and aerodynamic forces, thereby resulting in larger fiber diameters. Although solvent evaporation plays a role in fiber solidification, it is not the primary factor controlling fiber diameter. Incomplete evaporation may lead to slight fiber fusion or local swelling, but the dominant mechanism is viscosity-induced resistance to jet thinning [32]. The addition of erythritol increases the total mass fraction of solids [33], leading to an increase in the viscosity and surface tension of the spinning solution [34], as shown in Figure 3i. The viscosity effect in the solution [6] plays a leading role, increasing the entanglement degree between polymer chains, resisting external driving forces, and increasing the average diameter of the fibers. The diameter increases from 0.272 μm for 50% Ery-loaded fibers to 0.541 μm for 80% Ery-loaded fibers. The increase in surface tension also hinders the stretching of the spinning solution into fibers [35]. The solution cannot be stretched in time, forming beads, and the beads are elongated at the edges, forming spindle-shaped beaded fibers [33], as shown in Figure 3d. This collar or bead-on-string morphology has been widely reported in electrospinning and solution blow spinning systems. It is generally attributed to Rayleigh instability, where insufficient viscoelastic force cannot counterbalance surface tension, leading to the breakup of the jet into droplets connected by thin filaments. With the increase in erythritol concentration, the number of beaded fibers greatly increases, as shown in Figure 3e. Simultaneously, the phenomenon of parallel merging and adhesion between adjacent fibers may be due to the incomplete evaporation of the solvent [36]. The coaxial fiber morphology is also smooth, cylindrical, and without fiber breakage, as shown in Figure 3f,g. No beaded fiber structure is observed, confirming the effective encapsulation of PEO-80%Ery fibers. In the core–shell structure, due to the coaxial configuration of two different types of organic molecules, the fiber diameter is thicker, with PVA/PEO-80%Ery at 0.666 μm and PVP/PEO-80%Ery at 0.66 μm, far greater than the diameter of PEO-80%Ery fibers (Figure 3h). However, compared to PVA/PEO-80%Ery fibers, the PVP/PEO-80%Ery fibers are discontinuous, prone to breakage, and have a poor morphology, which may lead to lower encapsulation. The statistical distribution and standard deviation of fiber diameters are presented in Figure 3h to provide a more comprehensive evaluation. The viscosity data shown in Figure 3i further confirm the correlation between solution properties and fiber morphology.

**Figure 4:**
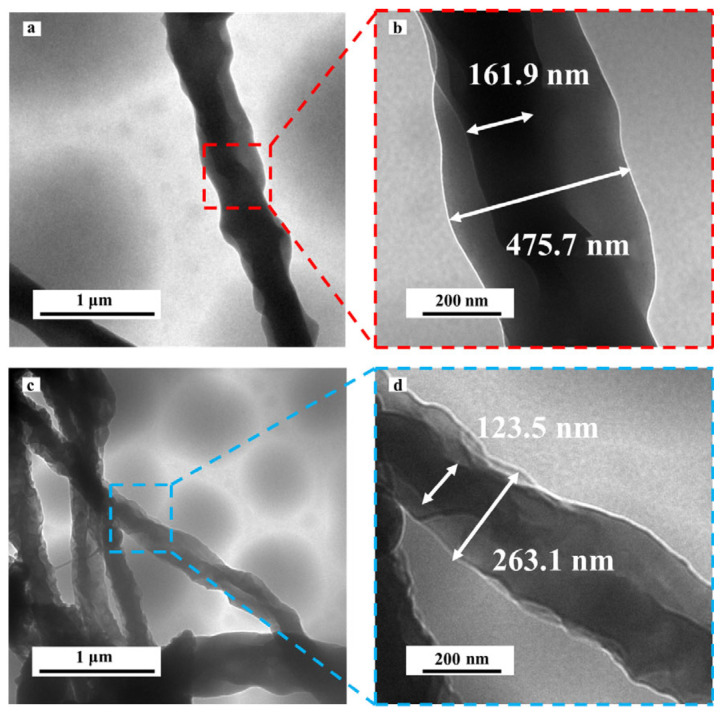
(**a**) TEM images of coaxial fibers. (**b**) TEM images of PVA/PEO-80% Ery, where (**b**) is a partial enlargement of (**a**). (**c**,**d**) TEM images of PVP/PEO-80% Ery, where (**d**) is a partial enlargement of (**c**).

In order to further observe the encapsulation in the fibers, the TEM maps of the coaxial fibers were compared, as shown in Figure 4. Due to the different materials between the core and shell of the fibers, distinct and clear boundaries were obtained through the electron density differences. As shown in Figure 4a,c, it indicates that PEO-80% Ery is completely encapsulated by PVA and PVP. The coaxial structure with PVA as the shell, possessing a core diameter of 161.9 nm and a shell diameter of 475.7 nm, can be obtained through Figure 4b. The coaxial structure with PVP as the shell, with a core diameter of 123.5 nm and a shell diameter of 263.1 nm, can be obtained through Figure 4d. PEO-80%Ery can be stably encapsulated in the PVA, PVP shell. The formation of a stable core–shell structure is also closely related to the rheological properties of both core and shell solutions, where appropriate viscosity ensures continuous jet formation without breakup. Therefore, the coaxial structure was successfully prepared to alleviate the problem of erythritol leakage.

### 3.3. Thermal Storage Properties of Fibers

**Figure 5:**
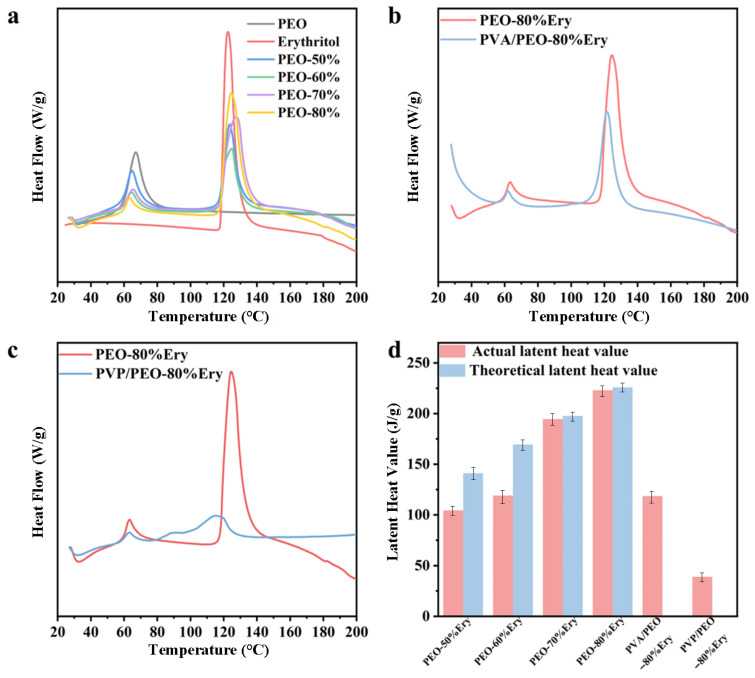
DSC testing curves of the fibers: (**a**) PEO, erythritol, and PEO-Ery composite fibers; (**b**) PVA/PEO-80%Ery coaxial fibers; (**c**) PVP/PEO-80%Ery coaxial fibers. (**d**) Comparison between the actual latent heat of the fibers and the theoretical latent heat.

The composite fibers exhibit two peaks, as shown in Figure 5a, with the peak between 60 and 70 °C corresponding to the endothermic peak of PEO, and the peak between 117 and 120 °C corresponding to the endothermic peak of erythritol. The phase transition temperature of all composite fibers shows no significant change compared to PEO and erythritol. The slight decrease may be attributed to the weaker molecular interactions between erythritol and PEO, which affect the energy required for phase transition and result in a decrease in the phase transition temperature [37]. The presence of peaks at the same positions in the PEO fiber structure indicates the successful loading of erythritol in PEO. The main function of PEO is to load erythritol, so the focus is no longer on the latent heat value of PEO. The latent heat of the composite fiber depends on the mass ratio of erythritol and increases with increasing erythritol concentration, increased from 104.41 J/g at 50% Ery loading to 223.01 J/g at 80% Ery loading. The theoretical latent heat value of the composite fiber is obtained by multiplying the latent heat of the PCM with the mass percentage of the PCM in the composite fiber [38]. It is evident from Figure 5d that the latent heat values of all composite fibers are lower than their corresponding theoretical values, with latent heat efficiency (the ratio of experimental value to theoretical value) less than 100%. The deviation between experimental and theoretical values may be due to the hindered thermal transition of erythritol caused by the movement of PEO molecules during phase transition [39], resulting in a decrease in crystallinity and latent heat of erythritol in the composite fiber. As the erythritol content increases, the variation in the difference between the theoretical and actual values decreases, probably due to the fact that the hindering effect of PEO is attenuated by the large amount of erythritol.

The coaxial thermal storage fiber also exhibits two exothermic peaks, as shown in Figure 5b,c with a significant overlap in the phase transition range with the composite fiber encapsulated in the core, indicating similar thermal behavior. PVA/PEO-80%Ery the latent heat of phase change is 118.63 J/g. The shell material PVA has no effect at temperatures below 200 °C, confirming the successful preparation of the coaxial fiber. The latent heat of phase transition of PVP/PEO-80% Ery was 38.997 J/g, much smaller than PVA/PEO-80% Ery, as shown in Table 1, with poor thermal properties.

The encapsulation efficiency of the coaxial fiber is calculated using the following Formula (2) [40].

(2) $$\varphi =\frac{∆HCPCM}{∆Hpure}\times 100\%$$

where φ is the encapsulation efficiency, ∆HCPCM is the latent heat of phase transition of the coaxial spinning fiber, and ∆Hpure is the latent heat of phase transition of PEO-80%Ery. After calculation, we determined the encapsulation efficiency of PVA/PEO-80%Ery to be 53%. The encapsulation rate of PVP/PEO-80%Ery was only 17.4%. The DSC results showed that the PVA/PEO-80%Ery fibers structure had a high latent heat of phase transition and encapsulation rate, as well as better thermal properties.

### 3.4. Thermal Stability of Fibers

**Figure 6:**
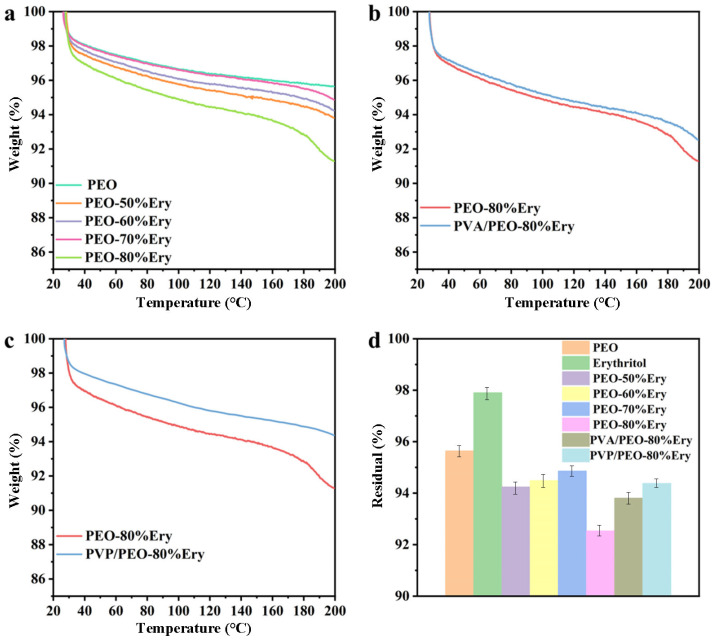
The TGA test curves of the fibers: (**a**) PEO, erythritol, and PEO-Ery composite fibers; (**b**) PVA/PEO-80%Ery coaxial fibers; (**c**) PVP/PEO-80%Ery coaxial fibers. (**d**) Residual rate of the fibers.

The TGA curves of the fibers all show the same trend. As shown in Figure 6a, the weight loss between 30 and 40 °C is due to the decomposition of PEO and the volatilization of residual solvents in the fiber. As the temperature increases, the amount of weight loss decreases and tends to stabilize. Even at 200 °C, the residual rate can still be maintained at over 90%, as shown in Figure 6d. The residual rates of the coaxial fibers PVA/PEO-80%Ery and PVP/PEO-80%Ery are both higher than that of PEO-80%Ery, as shown in Figure 6b,c. The residual rates are 93.81% and 94.38%, as shown in Table 2. This indicates that the coaxial fibers have relatively good thermal stability.

## 4. Conclusions

In summary, PEO-Ery composite fibers as well as PVA/PEO-80%Ery and PVP/PEO-80%Ery coaxial fibers were prepared by electro-blown spinning. The results showed that the composite fibers were prepared without the generation of new substances, the fibers exhibited a predominantly cylindrical morphology based on SEM observations, and the diameters increased with the increase in the content of erythritol. The diameter of the pure PEO fiber was 0.158 μm, and the diameter of the composite fiber loaded with 80% Ery was 0.541 μm. At the same time, a large number of spindle-like beaded fibers appeared in the fibers with high loading capacity. The latent heat also increased with the increase in erythritol content, from 104.41 J/g for 50% Ery-loaded composite fibers to 223.01 J/g for 80% Ery-loaded composite fibers. Coaxial fiber is smooth, and no spindle-like beaded fibers appear. TEM results exhibited an obvious core–shell structure, indicating that PVA successfully encapsulates PEO-80% Ery. PVA/PEO-80% Ery coaxial fibers had a high latent heat of 118.62 J/g and a high encapsulation rate of 53%, much higher than that of PVP/PEO-80% Ery. The problem of erythritol leakage has been solved. The TGA results showed that the fibers maintained good stability in the phase transition temperature range. The research proposal of encapsulating PEO-Ery with PVA provides valuable guidance for the preparation and industrialization of erythritol thermal storage fibers.

## Figures and Tables

**Table 1:** Thermal property data for PEO, erythritol, and PEO-Ery composite fibers, and PVA/PEO-80% Ery and PVP/PEO-80% Ery coaxial fibers.

Material	Phase Transition Temperature (°C)	Actual Latent Heat Value (J/g)	Theoretical Latent Heat Value (J/g)
PEO	67.1 ± 0.2	95.6 ± 1.5	
Erythritol	118.0 ± 0.4	282.3 ± 3.2	
PEO-50%Ery	117.9 ± 0.5	104.4 ± 1.7	141.1 ± 1.5
PEO-60%Ery	117.9 ± 0.4	119.0 ± 1.8	169.4 ± 1.9
PEO-70%Ery	118.3 ± 0.5	194.5 ± 2.1	197.6 ± 2.1
PEO-80%Ery	118.2 ± 0.5	223.0 ± 2.4	225.8 ± 2.3
PVA/PEO-80%Ery	116.2 ± 0.8	118.6 ± 1.8	
PVP/PEO-80%Ery	115.0 ± 0.4	39.0 ± 0.6	

**Table 2:** Thermal stability data for PEO, erythritol, and PEO-Ery composite fibers and PVA/PEO-80% Ery and PVP/PEO-80% Ery coaxial fibers.

Material	Loss Rate (%)	Residual Rate (%)
PEO	4.4 ± 0.2	95.6 ± 0.8
Erythritol	2.1 ± 0.1	97.9 ± 0.9
PEO-50%Ery	5.8 ± 0.2	94.2 ± 0.8
PEO-60%Ery	5.5 ± 0.2	94.5 ± 0.8
PEO-70%Ery	5.1 ± 0.2	94.9 ± 0.8
PEO-80%Ery	7.5 ± 0.3	92.5 ± 0.7
PVA/PEO-80%Ery	6.2 ± 0.2	93.8 ± 0.7
PVP/PEO-80%Ery	5.7 ± 0.2	94.4 ± 0.8

## Data Availability

The original contributions presented in this study are included in the article. Further inquiries can be directed to the corresponding authors.

## References

[1] Du Y., Ding Y. (2016). Towards improving charge/discharge rate of latent heat thermal energy storage (LHTES) by embedding metal foams in phase change materials (PCMs). Chem. Eng. Process. Process Intensif..

[2] Babapoor A., Karimi G., Sabbaghi S. (2016). Thermal characteristic of nanocomposite phase change materials during solidification process. J. Energy Storage.

[3] Zgardzińska B., Filipek M., Fortuniak W., Mroczek P. (2016). Properties of n-eicosane-filled microcapsules with different morphology. Phase Change Materials studied by positron spectroscopy and complementary methods. Mater. Chem. Phys..

[4] Yuan M., Xu C., Wang T., Zhang T., Pan X., Ye F. (2021). Supercooling suppression and crystallization behaviour of erythritol/expanded graphite as form-stable phase change material. Chem. Eng. J..

[5] Wang F., Nasajpour-Esfahani N., Alizadeh A.A., Smaisim G.F., Abed A.M., Hadrawi S.K., Aminian S., Sabetvand R., Toghraie D. (2023). Thermal performance of a phase change material (PCM) microcapsules containing Au nanoparticles in a nanochannel: A molecular dynamics approach. J. Mol. Liq..

[6] Kizildag N. (2021). Smart composite nanofiber mats with thermal management functionality. Sci. Rep..

[7] Li L., Liu X., Wang G., Liu Y., Kang W., Deng N., Zhuang X., Zhou X. (2021). Research progress of ultrafine alumina fiber prepared by sol-gel method: A review. Chem. Eng. J..

[8] Tong H.-W., Wang M. (2013). A novel technique for the fabrication of 3D nanofibrous scaffolds using simultaneous positive voltage electrospinning and negative voltage electrospinning. Mater. Lett..

[9] Bhardwaj N., Kundu S.C. (2010). Electrospinning: A fascinating fiber fabrication technique. Biotechnol. Adv..

[10] Daristotle J.L., Behrens A.M., Sandler A.D., Kofinas P. (2016). A review of the fundamental principles and applications of solution blow spinning. ACS Appl. Mater. Interfaces.

[11] Wang H., Liao S., Bai X., Liu Z., Fang M., Liu T., Wang N., Wu H. (2016). Highly flexible indium tin oxide nanofiber transparent electrodes by blow spinning. ACS Appl. Mater. Interfaces.

[12] Gao Y., Zhang J., Su Y., Wang H., Wang X.-X., Huang L.-P., Yu M., Ramakrishna S., Long Y.-Z. (2021). Recent progress and challenges in solution blow spinning. Mater. Horiz..

[13] Zou W., Chen R.Y., Zhang G.Z., Zhang H.C., Qu J.P. (2014). Recent advances in centrifugal spinning preparation of nanofibers. Adv. Mater. Res..

[14] Hu X., Zhang X., Shen X., Li H., Takai O., Saito N. (2014). Plasma-induced synthesis of CuO nanofibers and ZnO nanoflowers in water. Plasma Chem. Plasma Process..

[15] Medeiros E.S., Glenn G.M., Klamczynski A.P., Orts W.J., Mattoso L.H. (2009). Solution blow spinning: A new method to produce micro-and nanofibers from polymer solutions. J. Appl. Polym. Sci..

[16] Igor M., Ekaterina P., Markel V., Yaroslav G., Sergey L., Petr G., Georgy M., Natalia A., Denis K., Radmir G. (2025). Characterization of structure and morphology of cellulose lyocell microfibers extracted from PAN matrix. Polysaccharides.

[17] Zhuang X., Yang X., Shi L., Cheng B., Guan K., Kang W. (2012). Solution blowing of submicron-scale cellulose fibers. Carbohydr. Polym..

[18] Lou H., Han W., Wang X. (2014). Numerical study on the solution blowing annular jet and its correlation with fiber morphology. Ind. Eng. Chem. Res..

[19] Metera A. (2013). The Production of Polymer Fibres by Soulition Blow Spinning.

[20] Lou H., Li W., Li C., Wang X. (2013). Systematic investigation on parameters of solution blown micro/nanofibers using response surface methodology based on box-Behnken design. J. Appl. Polym. Sci..

[21] Atif R., Khaliq J., Combrinck M., Hassanin A.H., Shehata N., Elnabawy E., Shyha I. (2020). Solution blow spinning of polyvinylidene fluoride based fibers for energy harvesting applications: A review. Polymers.

[22] Cao Z., Zheng X., Qu Q., Huang Y., Zheng H. (2021). Electrolyte Design Enabling a High-Safety and High-Performance Si Anode with a Tailored Electrode–Electrolyte Interphase. Adv. Mater..

[23] Zhao Y.-X., Zhou X.-H., Li L., Xu W., Kang W.-M., Cheng B.-W. (2016). Preparation of porous CeO_2_/CuO/Al_2_O_3_ fibers via electro-blown spinning method. Mater. Lett..

[24] Sóti P.L., Bocz K., Pataki H., Eke Z., Farkas A., Verreck G., Kiss É., Fekete P., Vigh T., Wagner I. (2015). Comparison of spray drying, electroblowing and electrospinning for preparation of Eudragit E and itraconazole solid dispersions. Int. J. Pharm..

[25] Ju J., Kang W., Deng N., Li L., Zhao Y., Ma X., Fan L., Cheng B. (2017). Preparation and characterization of PVA-based carbon nanofibers with honeycomb-like porous structure via electro-blown spinning method. Microporous Mesoporous Mater..

[26] Liu Y., Jia C., Zhang H., Wang H., Li P., Jia L., Wang F., Zhu P., Wang H., Yu L. (2021). Free-standing ultrafine nanofiber papers with high PM0. 3 mechanical filtration efficiency by scalable blow and electro-blow spinning. ACS Appl. Mater. Interfaces.

[27] Sun S.-X., Xie R., Wang X.-X., Wen G.-Q., Liu Z., Wang W., Ju X.-J., Chu L.-Y. (2015). Fabrication of nanofibers with phase-change core and hydrophobic shell, via coaxial electrospinning using nontoxic solvent. J. Mater. Sci..

[28] Xu H., Yagi S., Ashour S., Du L., Hoque M.E., Tan L. (2023). A Review on Current Nanofiber Technologies: Electrospinning, Centrifugal Spinning, and Electro-Centrifugal Spinning. Macromol. Mater. Eng..

[29] Lu Y., Xiao X., Fu J., Huan C., Qi S., Zhan Y., Zhu Y., Xu G. (2019). Novel smart textile with phase change materials encapsulated core-sheath structure fabricated by coaxial electrospinning. Chem. Eng. J..

[30] Hu W., Yu X. (2012). Encapsulation of bio-based PCM with coaxial electrospun ultrafine fibers. Rsc Adv..

[31] Zhou X.-h., Kang W.-m., Xu W., Cheng B.-w. (2015). Flexible hollow CeO_2_/Al_2_O_3_ fibers: Preparation, characterization and dye adsorption efficiency. RSC Adv..

[32] Semnani Rahbar R., Maleki H., Kalantari B. (2016). Fabrication of electrospun nanofibre yarn based on nylon 6/microencapsulated phase change materials. J. Exp. Nanosci..

[33] Zhang X., Li Q., Holesinger T.G., Arendt P.N., Huang J., Kirven P.D., Clapp T.G., DePaula R.F., Liao X., Zhao Y. (2007). Ultrastrong, stiff, and lightweight carbon-nanotube fibers. Adv. Mater..

[34] De P., Sathyanarayana D., Sadasivamurthy P., Sridhar S. (2002). Reactivity ratios for the oxidative copolymerizations of indene with methyl methacrylate and methacrylonitrile. Eur. Polym. J..

[35] Babapoor A., Karimi G., Golestaneh S.I., Mezjin M.A. (2017). Coaxial electro-spun PEG/PA6 composite fibers: Fabrication and characterization. Appl. Therm. Eng..

[36] Jiang L., Lu Y., Liu X., Tu H., Zhang J., Shi X., Deng H., Du Y. (2015). Layer-by-layer immobilization of quaternized carboxymethyl chitosan/organic rectorite and alginate onto nanofibrous mats and their antibacterial application. Carbohydr. Polym..

[37] Zhang D., Tian S., Xiao D. (2007). Experimental study on the phase change behavior of phase change material confined in pores. Sol. Energy.

[38] Chen C., Wang L., Huang Y. (2008). A novel shape-stabilized PCM: Electrospun ultrafine fibers based on lauric acid/polyethylene terephthalate composite. Mater. Lett..

[39] Seifpoor M., Nouri M., Mokhtari J. (2011). Thermo-regulating nanofibers based on nylon 6, 6/polyethylene glycol blend. Fibers Polym..

[40] Huang X., Chen X., Li A., Atinafu D., Gao H., Dong W., Wang G. (2019). Shape-stabilized phase change materials based on porous supports for thermal energy storage applications. Chem. Eng. J..

